# Supplementary figures and images for: The occurrence of ‘Sleeping Beauty’ publications in medical research: Their scientific impact and technological relevance
Source: PLoS One. 2019 Oct 18;14(10):e0223373. doi: 10.1371/journal.pone.0223373 (PMC6799932; doi:10.1371/journal.pone.0223373)

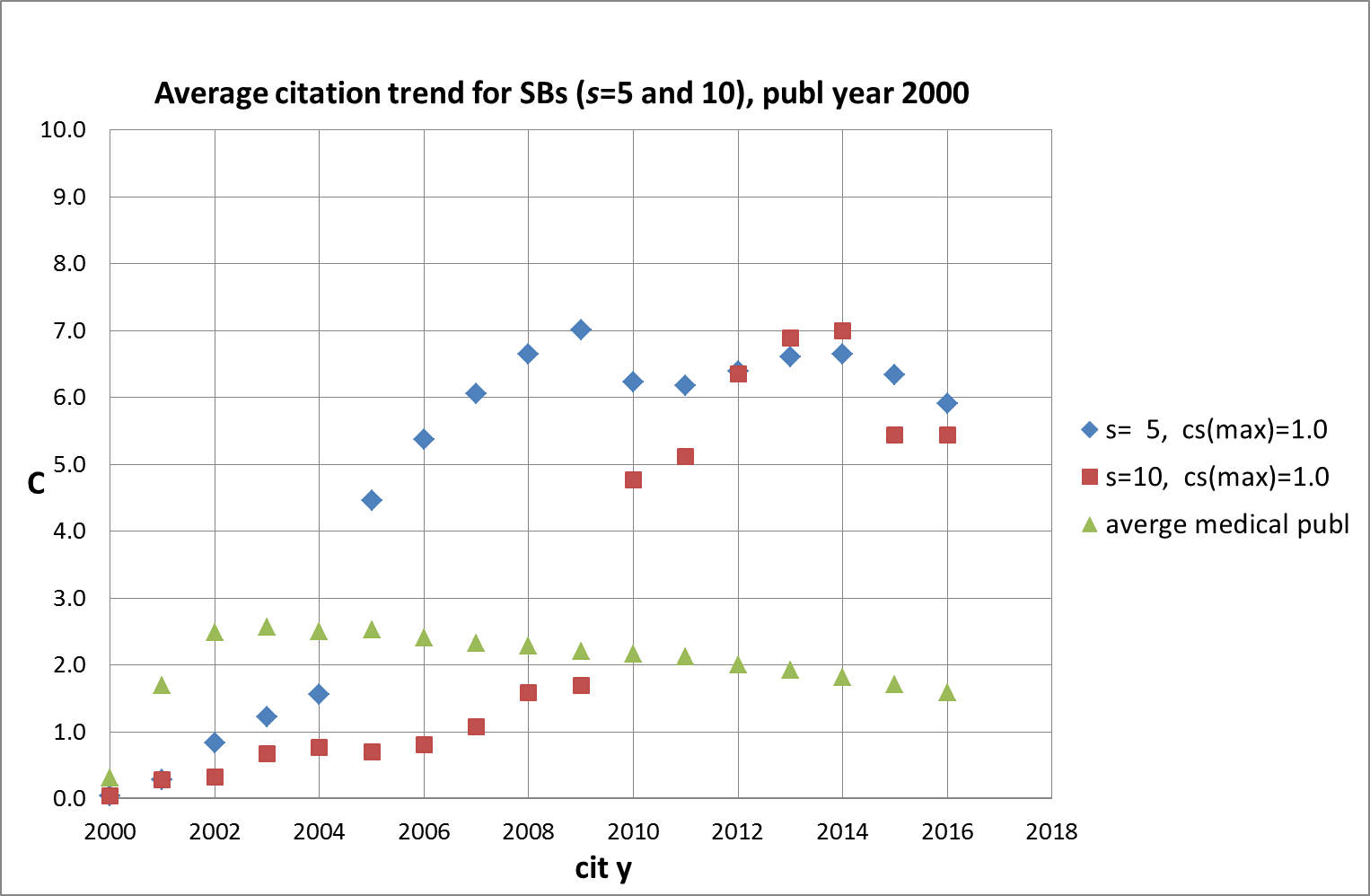

Supplement: S1 Fig — (TIF) [file pone.0223373.s001.tif]

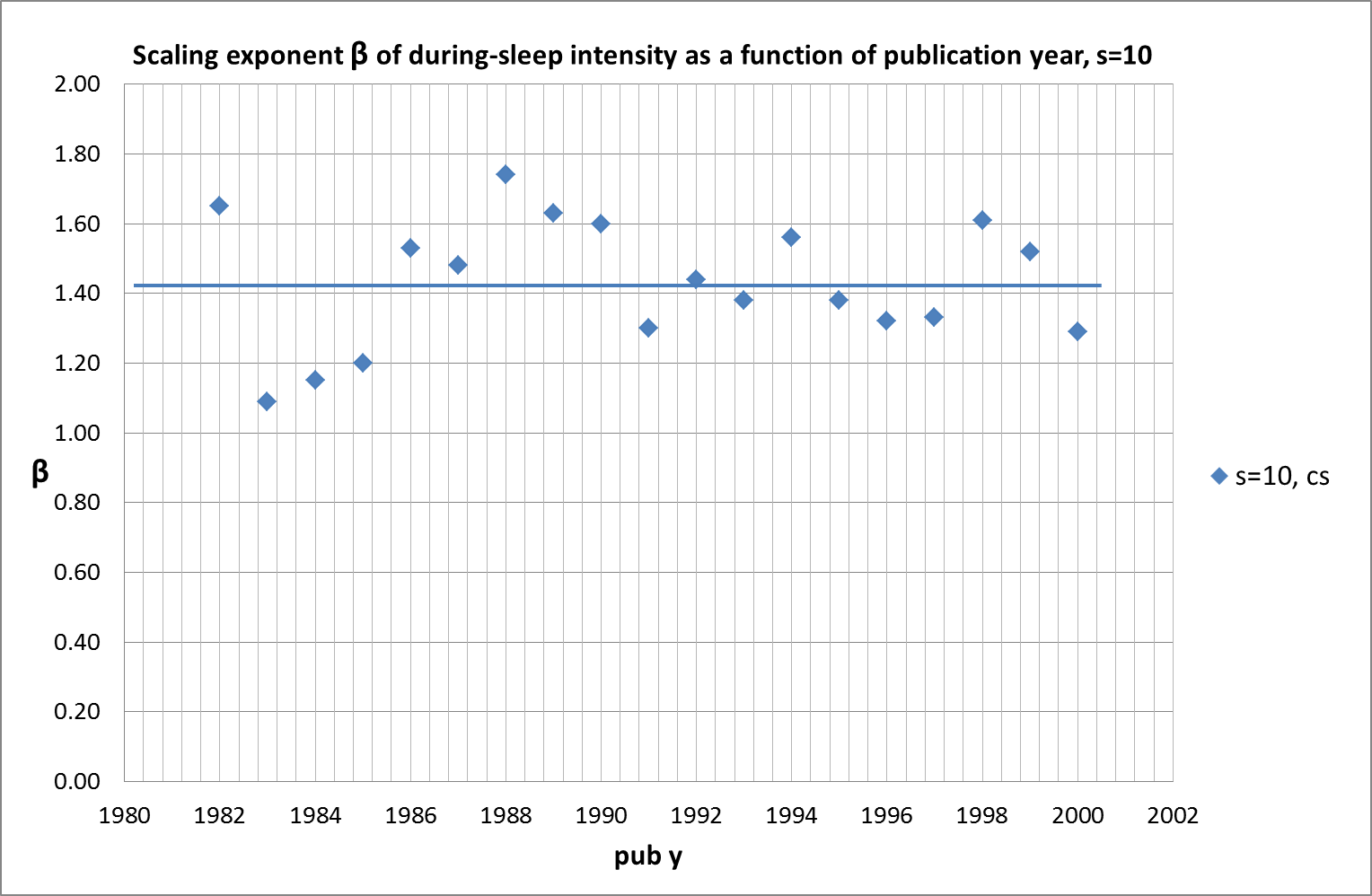

Supplement: S2 Fig — (TIF) [file pone.0223373.s002.tif]

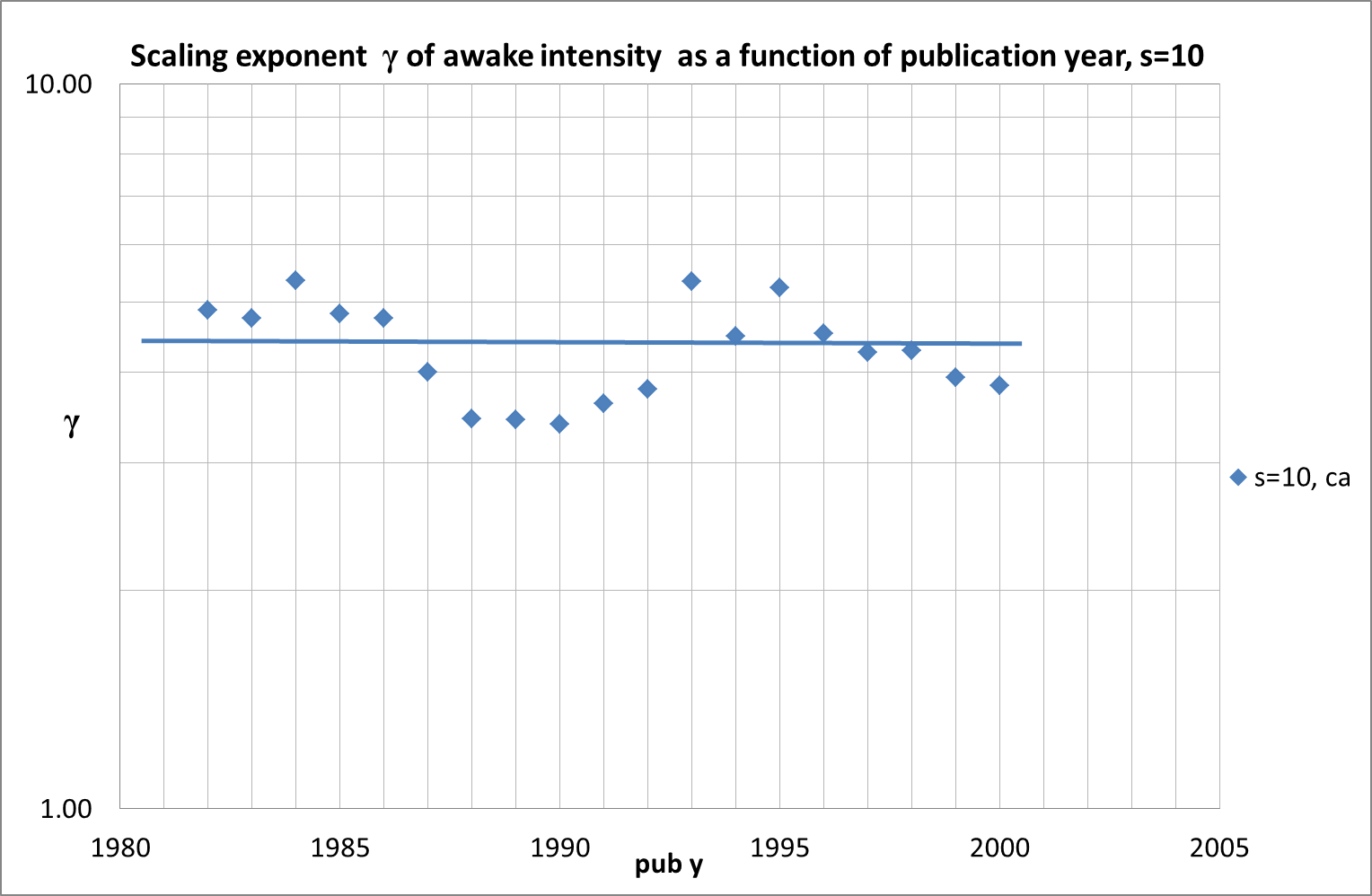

Supplement: S3 Fig — (TIF) [file pone.0223373.s003.tif]
