# Supplementary material for: The occurrence of ‘Sleeping Beauty’ publications in medical research: Their scientific impact and technological relevance
Source: PLoS One. 2019 Oct 18;14(10):e0223373. doi: 10.1371/journal.pone.0223373 (PMC6799932; doi:10.1371/journal.pone.0223373)
Supplement: S1 Table — (DOCX) [file pone.0223373.s004.docx]

**S1 Table. WoS Fields codes and names of medical research fields.**

| **Clinical Medicine** |  |  |  |  |  |  |
| --- | --- | --- | --- | --- | --- | --- |
| **WoS field code and name** | |  |  |  |  |  |
| 9              ALLERGY |  |  |  |  |  |  |
| 10           ANATOMY & MORPHOLOGY | | |  |  |  |  |
| 11           ANDROLOGY | |  |  |  |  |  |
| 12           ANESTHESIOLOGY | |  |  |  |  |  |
| 32           ONCOLOGY | |  |  |  |  |  |
| 33           CARDIAC & CARDIOVASCULAR SYSTEMS | | | |  |  |  |
| 56           EMERGENCY MEDICINE | | |  |  |  |  |
| 61           DENTISTRY/ORAL SURGERY & MEDICINE | | | |  |  |  |
| 62           DERMATOLOGY | |  |  |  |  |  |
| 74           ENDOCRINOLOGY & METABOLISM | | | |  |  |  |
| 99           GASTROENTEROLOGY & HEPATOLOGY | | | |  |  |  |
| 100         GENETICS & HEREDITY | | |  |  |  |  |
| 105         GERIATRICS & GERONTOLOGY | | |  |  |  |  |
| 106         HEALTH POLICY & SERVICES | | |  |  |  |  |
| 107         HEMATOLOGY | |  |  |  |  |  |
| 113         PUBLIC, ENVIRONMENTAL & OCCUPATIONAL HEALTH | | | | | | |
| 114         IMMUNOLOGY | |  |  |  |  |  |
| 116         INFECTIOUS DISEASES | | |  |  |  |  |
| 122         MEDICINE, LEGAL | |  |  |  |  |  |
| 143         MEDICAL INFORMATICS | | |  |  |  |  |
| 146         MEDICINE, GENERAL & INTERNAL | | | |  |  |  |
| 147         METALLURGY & METALLURGICAL ENGINEERING | | | | |  |  |
| 148         MEDICINE, RESEARCH & EXPERIMENTAL | | | |  |  |  |
| 166         CLINICAL NEUROLOGY | | |  |  |  |  |
| 167         NEUROSCIENCES | |  |  |  |  |  |
| 169         NURSING |  |  |  |  |  |  |
| 170         NUTRITION & DIETETICS | | |  |  |  |  |
| 171         OBSTETRICS & GYNECOLOGY | | |  |  |  |  |
| 174         OPHTHALMOLOGY | |  |  |  |  |  |
| 177         ORTHOPEDICS | |  |  |  |  |  |
| 178         OTORHINOLARYNGOLOGY | | |  |  |  |  |
| 180         PARASITOLOGY | |  |  |  |  |  |
| 181         PATHOLOGY | |  |  |  |  |  |
| 182         PEDIATRICS | |  |  |  |  |  |
| 183         PHARMACOLOGY & PHARMACY | | |  |  |  |  |
| 199         PSYCHIATRY | |  |  |  |  |  |
| 206         RADIOLOGY, NUCLEAR MEDICINE & MEDICAL IMAGING | | | | | | |
| 207         REHABILITATION | |  |  |  |  |  |
| 208         RESPIRATORY SYSTEM | | |  |  |  |  |
| 209         REPRODUCTIVE BIOLOGY | | |  |  |  |  |
| 210         RHEUMATOLOGY | |  |  |  |  |  |
| 214         SOCIAL SCIENCES, BIOMEDICAL | | |  |  |  |  |
| 219         SPORT SCIENCES | |  |  |  |  |  |
| 221         SURGERY |  |  |  |  |  |  |
| 225         TOXICOLOGY | |  |  |  |  |  |
| 226         TRANSPLANTATION | |  |  |  |  |  |
| 228         TROPICAL MEDICINE | |  |  |  |  |  |
| 230         UROLOGY & NEPHROLOGY | | |  |  |  |  |
| 232         PERIPHERAL VASCULAR DISEASE | | | |  |  |  |
| 233         VIROLOGY |  |  |  |  |  |  |
| 241         HEALTH CARE SCIENCES & SERVICES | | | |  |  |  |
| 245         CRITICAL CARE MEDICINE | | |  |  |  |  |
| 248         INTEGRATIVE & COMPLEMENTARY MEDICINE | | | | |  |  |
| 249         NEUROIMAGING | |  |  |  |  |  |
| 250         GERONTOLOGY | |  |  |  |  |  |
| 256         MEDICAL ETHICS | |  |  |  |  |  |
| 258         PRIMARY HEALTH CARE | | |  |  |  |  |
| 259         AUDIOLOGY & SPEECH-LANGUAGE PATHOLOGY | | | | |  |  |
