# Supplementary material for: The occurrence of ‘Sleeping Beauty’ publications in medical research: Their scientific impact and technological relevance
Source: PLoS One. 2019 Oct 18;14(10):e0223373. doi: 10.1371/journal.pone.0223373 (PMC6799932; doi:10.1371/journal.pone.0223373)
Supplement: S2 Table — (DOCX) [file pone.0223373.s005.docx]

**S2 Table. Numbers of the identified SBs**.

|  | ***c_s_*(max)** | |  |  |  |
| --- | --- | --- | --- | --- | --- |
| **s=5** | **0.2** | **0.4** | **0.6** | **0.8** | **1.0** |
| 1980 | 3 | 6 | 15 | 28 | 52 |
| 1981 | 5 | 11 | 18 | 32 | 55 |
| 1982 | 1 | 6 | 14 | 33 | 56 |
| 1983 | 4 | 10 | 19 | 35 | 66 |
| 1984 | 3 | 7 | 22 | 35 | 62 |
| 1985 | 2 | 9 | 27 | 37 | 73 |
| 1986 | 8 | 18 | 31 | 50 | 87 |
| 1987 | 4 | 13 | 41 | 76 | 122 |
| 1988 | 5 | 20 | 44 | 78 | 133 |
| 1989 | 10 | 28 | 55 | 107 | 176 |
| 1990 | 14 | 33 | 66 | 127 | 213 |
| 1991 | 21 | 49 | 95 | 160 | 252 |
| 1992 | 15 | 30 | 62 | 109 | 196 |
| 1993 | 8 | 22 | 47 | 91 | 152 |
| 1994 | 6 | 16 | 48 | 75 | 123 |
| 1995 | 3 | 13 | 42 | 84 | 140 |
| 1996 | 14 | 27 | 52 | 93 | 168 |
| 1997 | 10 | 28 | 57 | 108 | 211 |
| 1998 | 14 | 37 | 76 | 127 | 224 |
| 1999 | 15 | 47 | 98 | 185 | 300 |
| 2000 | 14 | 38 | 95 | 208 | 343 |
| 2001 | 13 | 36 | 91 | 194 | 350 |
| 2002 | 11 | 44 | 100 | 190 | 351 |
| 2003 | 6 | 27 | 78 | 169 | 316 |
| 2004 | 10 | 32 | 78 | 169 | 302 |
| 2005 | 12 | 27 | 52 | 113 | 249 |
| 2006 | 7 | 24 | 62 | 124 | 231 |
| 2007 | 11 | 25 | 64 | 125 | 244 |

|  | ***c_s_*(max)** | |  |  |  |
| --- | --- | --- | --- | --- | --- |
| **s=10** | **0.2** | **0.4** | **0.6** | **0.8** | **1.0** |
| 1980 | 0 | 0 | 5 | 8 | 13 |
| 1981 | 0 | 0 | 1 | 2 | 9 |
| 1982 | 0 | 0 | 3 | 4 | 10 |
| 1983 | 1 | 5 | 6 | 8 | 14 |
| 1984 | 0 | 3 | 4 | 13 | 29 |
| 1985 | 3 | 7 | 15 | 23 | 38 |
| 1986 | 0 | 6 | 14 | 25 | 38 |
| 1987 | 0 | 2 | 5 | 11 | 25 |
| 1988 | 0 | 0 | 4 | 8 | 25 |
| 1989 | 1 | 1 | 1 | 5 | 17 |
| 1990 | 1 | 2 | 9 | 13 | 18 |
| 1991 | 0 | 3 | 7 | 11 | 17 |
| 1992 | 0 | 3 | 5 | 12 | 23 |
| 1993 | 1 | 2 | 2 | 4 | 12 |
| 1994 | 0 | 2 | 3 | 9 | 24 |
| 1995 | 0 | 3 | 7 | 17 | 31 |
| 1996 | 0 | 1 | 3 | 13 | 34 |
| 1997 | 1 | 3 | 11 | 24 | 43 |
| 1998 | 2 | 3 | 5 | 21 | 46 |
| 1999 | 0 | 5 | 10 | 23 | 48 |
| 2000 | 1 | 4 | 8 | 21 | 43 |
| 2001 | 1 | 2 | 5 | 15 | 32 |
| 2002 | 3 | 4 | 7 | 15 | 25 |

|  | ***c_s_*(max)** | |  |  |  |
| --- | --- | --- | --- | --- | --- |
| **s=15** | **0.2** | **0.4** | **0.6** | **0.8** | **1.0** |
| 1980 | 1 | 1 | 1 | 1 | 7 |
| 1981 | 0 | 1 | 1 | 5 | 10 |
| 1982 | 0 | 0 | 2 | 11 | 15 |
| 1983 | 2 | 2 | 2 | 5 | 12 |
| 1984 | 0 | 0 | 1 | 2 | 5 |
| 1985 | 2 | 2 | 3 | 4 | 7 |
| 1986 | 0 | 0 | 2 | 6 | 8 |
| 1987 | 1 | 2 | 2 | 4 | 7 |
| 1988 | 0 | 0 | 1 | 4 | 8 |
| 1989 | 0 | 1 | 2 | 8 | 11 |
| 1990 | 0 | 0 | 2 | 6 | 14 |
| 1991 | 0 | 0 | 5 | 7 | 13 |
| 1992 | 0 | 0 | 1 | 5 | 11 |
| 1993 | 0 | 0 | 2 | 4 | 10 |
| 1994 | 0 | 2 | 4 | 7 | 11 |
| 1995 | 1 | 3 | 6 | 10 | 17 |
| 1996 | 0 | 2 | 5 | 7 | 17 |
| 1997 | 1 | 1 | 4 | 10 | 16 |

|  | ***c_s_*(max)** | |  |  |  |
| --- | --- | --- | --- | --- | --- |
| **s=20** | **0.2** | **0.4** | **0.6** | **0.8** | **1.0** |
| 1980 | 0 | 0 | 1 | 2 | 3 |
| 1981 | 0 | 0 | 1 | 3 | 4 |
| 1982 | 0 | 0 | 1 | 2 | 4 |
| 1983 | 1 | 1 | 2 | 3 | 5 |
| 1984 | 0 | 1 | 1 | 4 | 10 |
| 1985 | 0 | 1 | 4 | 6 | 11 |
| 1986 | 0 | 0 | 0 | 3 | 6 |
| 1987 | 0 | 1 | 1 | 5 | 11 |
| 1988 | 0 | 0 | 1 | 5 | 10 |
| 1989 | 0 | 2 | 3 | 4 | 14 |
| 1990 | 2 | 3 | 3 | 5 | 9 |
| 1991 | 1 | 1 | 3 | 7 | 12 |
| 1992 | 0 | 1 | 2 | 7 | 11 |

|  | ***c_s_*(max)** | |  |  |  |
| --- | --- | --- | --- | --- | --- |
| **s=25** | **0.2** | **0.4** | **0.6** | **0.8** | **1.0** |
| 1980 | 0 | 3 | 3 | 6 | 11 |
| 1981 | 0 | 0 | 0 | 1 | 2 |
| 1982 | 0 | 2 | 4 | 5 | 8 |
| 1983 | 0 | 1 | 1 | 4 | 6 |
| 1984 | 1 | 2 | 3 | 5 | 8 |
| 1985 | 0 | 1 | 4 | 7 | 10 |
| 1986 | 0 | 1 | 3 | 5 | 11 |
| 1987 | 0 | 0 | 1 | 3 | 6 |

|  | ***c_s_*(max)** | |  |  |  |
| --- | --- | --- | --- | --- | --- |
| **s=30** | **0.2** | **0.4** | **0.6** | **0.8** | **1.0** |
| 1980 | 0 | 0 | 0 | 2 | 6 |
| 1981 | 0 | 0 | 2 | 3 | 9 |
| 1982 | 0 | 1 | 2 | 4 | 4 |
