# Supplementary material for: The occurrence of ‘Sleeping Beauty’ publications in medical research: Their scientific impact and technological relevance
Source: PLoS One. 2019 Oct 18;14(10):e0223373. doi: 10.1371/journal.pone.0223373 (PMC6799932; doi:10.1371/journal.pone.0223373)
Supplement: S3 Table — (DOCX) [file pone.0223373.s006.docx]

**S3 Table. Trend of real and normalized number of SBs with *s*=5 (*c_s_*(max)=1) in the medical research fields, numbers are based on successive five-years blocks.**

| ***s=5*** | ***not-normalized*** | ***normalized*** |
| --- | --- | --- |
| 1980-84 | 291 | 520 |
| 1981-85 | 312 | 529 |
| 1982-86 | 344 | 564 |
| 1983-87 | 410 | 651 |
| 1984-88 | 477 | 734 |
| 1985-89 | 591 | 869 |
| 1986-90 | 731 | 1,044 |
| 1987-91 | 896 | 1,244 |
| 1988-92 | 970 | 1,311 |
| 1989-93 | 989 | 1,319 |
| 1990-94 | 936 | 1,232 |
| 1991-95 | 863 | 1,106 |
| 1992-96 | 779 | 950 |
| 1993-97 | 794 | 923 |
| 1994-98 | 866 | 962 |
| 1995-99 | 1,043 | 1,110 |
| 1996-00 | 1,246 | 1,271 |
| 1997-01 | 1,428 | 1,442 |
| 1998-02 | 1,568 | 1,568 |
| 1999-03 | 1,660 | 1,660 |
| 2000-04 | 1,662 | 1,629 |
| 2001-05 | 1,568 | 1,493 |
| 2002-06 | 1,449 | 1,329 |
| 2003-07 | 1,342 | 1,167 |
| 2004-08 | 1,283 | 1.043 |
