# Supplementary material for: The occurrence of ‘Sleeping Beauty’ publications in medical research: Their scientific impact and technological relevance
Source: PLoS One. 2019 Oct 18;14(10):e0223373. doi: 10.1371/journal.pone.0223373 (PMC6799932; doi:10.1371/journal.pone.0223373)
Supplement: S4 Table — (DOCX) [file pone.0223373.s007.docx]

**S4 Table. Number of all publications from *s*=1 to *s*=20, with *c_s_*(max)=1, *c_a_*(min)=5, *a*(min)=*a*(max)=5, within period 1980-2008.**

| ***s*** | N | last publ y | #publ y |
| --- | --- | --- | --- |
| 1 | 501,671 | 2008 | 29 |
| 2 | 170,672 | 2008 | 29 |
| 3 | 39,903 | 2008 | 29 |
| 4 | 12,650 | 2008 | 29 |
| 5 | 5,508 | 2008 | 29 |
| 6 | 2,887 | 2007 | 28 |
| 7 | 1,718 | 2006 | 27 |
| 8 | 1,178 | 2005 | 26 |
| 9 | 858 | 2004 | 25 |
| 10 | 639 | 2003 | 24 |
| 11 | 505 | 2002 | 23 |
| 12 | 412 | 2001 | 22 |
| 13 | 345 | 2000 | 21 |
| 14 | 292 | 1999 | 20 |
| 15 | 215 | 1998 | 19 |
| 16 | 199 | 1997 | 18 |
| 17 | 178 | 1996 | 17 |
| 18 | 164 | 1995 | 16 |
| 19 | 124 | 1994 | 15 |
| 20 | 120 | 1993 | 14 |
