# Supplementary material for: The occurrence of ‘Sleeping Beauty’ publications in medical research: Their scientific impact and technological relevance
Source: PLoS One. 2019 Oct 18;14(10):e0223373. doi: 10.1371/journal.pone.0223373 (PMC6799932; doi:10.1371/journal.pone.0223373)
Supplement: S5 Table — (DOCX) [file pone.0223373.s008.docx]

**S5 Table. Number of SBs (*s*=5) for successive during-sleep citation-intensity intervals.**

| ***s=5*** | ***c_s_*** |  |  |  |  |  |
| --- | --- | --- | --- | --- | --- | --- |
| ***pub y*** | ***0.0*** | ***0.2*** | ***0.4*** | ***0.6*** | ***0.8*** | ***1.0*** |
| 1980-84 | 5 | 11 | 24 | 48 | 75 | 128 |
| 1981-85 | 4 | 11 | 29 | 57 | 72 | 140 |
| 1982-86 | 3 | 15 | 32 | 63 | 77 | 154 |
| 1983-87 | 4 | 17 | 36 | 83 | 93 | 177 |
| 1984-88 | 3 | 19 | 45 | 98 | 111 | 201 |
| 1985-89 | 2 | 27 | 59 | 110 | 150 | 243 |
| 1986-90 | 6 | 35 | 71 | 125 | 201 | 293 |
| 1987-91 | 11 | 43 | 89 | 158 | 247 | 348 |
| 1988-92 | 17 | 48 | 95 | 162 | 259 | 389 |
| 1989-93 | 19 | 49 | 94 | 163 | 269 | 395 |
| 1990-94 | 19 | 45 | 86 | 168 | 244 | 374 |
| 1991-95 | 16 | 37 | 77 | 164 | 225 | 344 |
| 1992-96 | 14 | 32 | 62 | 143 | 201 | 327 |
| 1993-97 | 8 | 33 | 65 | 140 | 205 | 343 |
| 1994-98 | 8 | 39 | 74 | 154 | 212 | 379 |
| 1995-99 | 10 | 46 | 96 | 173 | 272 | 446 |
| 1996-00 | 13 | 54 | 110 | 201 | 343 | 525 |
| 1997-01 | 12 | 54 | 120 | 231 | 405 | 606 |
| 1998-02 | 14 | 53 | 135 | 258 | 444 | 664 |
| 1999-03 | 14 | 45 | 133 | 270 | 484 | 714 |
| 2000-04 | 13 | 41 | 123 | 265 | 488 | 732 |
| 2001-05 | 13 | 39 | 114 | 233 | 436 | 733 |
| 2002-06 | 12 | 34 | 108 | 216 | 395 | 684 |
| 2003-07 | 14 | 32 | 89 | 199 | 366 | 642 |
