# Supplementary material for: The occurrence of ‘Sleeping Beauty’ publications in medical research: Their scientific impact and technological relevance
Source: PLoS One. 2019 Oct 18;14(10):e0223373. doi: 10.1371/journal.pone.0223373 (PMC6799932; doi:10.1371/journal.pone.0223373)
Supplement: S6 Table — (DOCX) [file pone.0223373.s009.docx]

**S6 Table. Number of SBs (*s*=10) for successive during-sleep citation-intensity intervals.**

|  |  | ***c_s_*** |  |  |  |  |  |  |  |  |  |
| --- | --- | --- | --- | --- | --- | --- | --- | --- | --- | --- | --- |
|  | ***s***=10 | ***0*** | ***0.2*** | ***0.3*** | ***0.4*** | ***0.5*** | ***0.6*** | ***0.7*** | ***0.8*** | ***0.9*** | ***1.0*** |
| 1980-84 | 1982 | 0 | 1 | 4 | 3 | 4 | 7 | 7 | 9 | 13 | 27 |
| 1981-85 | 1983 | 0 | 4 | 5 | 6 | 7 | 7 | 11 | 10 | 20 | 30 |
| 1982-86 | 1984 | 0 | 4 | 6 | 11 | 10 | 11 | 16 | 15 | 21 | 35 |
| 1983-87 | 1985 | 0 | 4 | 7 | 12 | 12 | 9 | 18 | 18 | 26 | 38 |
| 1984-88 | 1986 | 0 | 3 | 5 | 10 | 12 | 12 | 21 | 17 | 31 | 44 |
| 1985-89 | 1987 | 0 | 4 | 3 | 9 | 12 | 11 | 18 | 15 | 33 | 38 |
| 1986-90 | 1988 | 0 | 2 | 3 | 6 | 8 | 14 | 15 | 14 | 28 | 33 |
| 1987-91 | 1989 | 0 | 2 | 4 | 2 | 7 | 11 | 14 | 8 | 27 | 27 |
| 1988-92 | 1990 | 0 | 2 | 4 | 3 | 4 | 13 | 14 | 9 | 24 | 27 |
| 1989-93 | 1991 | 1 | 2 | 4 | 4 | 3 | 10 | 12 | 9 | 19 | 23 |
| 1990-94 | 1992 | 1 | 1 | 5 | 5 | 3 | 11 | 11 | 12 | 20 | 25 |
| 1991-95 | 1993 | 1 | 0 | 5 | 7 | 3 | 8 | 15 | 14 | 25 | 29 |
| 1992-96 | 1994 | 1 | 0 | 3 | 7 | 1 | 8 | 15 | 20 | 34 | 35 |
| 1993-97 | 1995 | 2 | 0 | 3 | 6 | 3 | 12 | 21 | 20 | 42 | 35 |
| 1994-98 | 1996 | 3 | 0 | 4 | 5 | 5 | 12 | 27 | 28 | 50 | 44 |
| 1995-99 | 1997 | 3 | 0 | 5 | 7 | 8 | 13 | 29 | 33 | 58 | 46 |
| 1996-00 | 1998 | 3 | 1 | 5 | 7 | 9 | 12 | 29 | 36 | 61 | 51 |
| 1997-01 | 1999 | 4 | 1 | 5 | 7 | 9 | 13 | 31 | 34 | 56 | 52 |
| 1998-02 | 2000 | 4 | 3 | 4 | 7 | 8 | 9 | 27 | 33 | 48 | 51 |
