# Supplementary material for: The occurrence of ‘Sleeping Beauty’ publications in medical research: Their scientific impact and technological relevance
Source: PLoS One. 2019 Oct 18;14(10):e0223373. doi: 10.1371/journal.pone.0223373 (PMC6799932; doi:10.1371/journal.pone.0223373)
Supplement: S7 Table — (DOCX) [file pone.0223373.s010.docx]

**S7 Table. Number of SBs (*s*=5) for awakening citation-intensity intervals.**

|  |  |  |  |  |  |  |  |  |
| --- | --- | --- | --- | --- | --- | --- | --- | --- |
|  | s=5 | 5.0≤ ***c_a_*** | 6.0< ***c_a_*** | 7.0< ***c_a_*** | 9.0<  ***c_a_*** | 11.0< ***c_a_*** | 13.0<  ***c_a_*** | 15.0<  ***c_a_*** |
|  |  | ≤6.0 | ≤7.0 | ≤9.0 | ≤11.0 | ≤13.0 | ≤15.0 | ≤17.0 |
| 1980-84 | 1982 | 191 | 40 | 41 | 11 | 4 | 1 | 2 |
| 1981-85 | 1983 | 203 | 47 | 41 | 13 | 4 | 1 | 2 |
| 1982-86 | 1984 | 220 | 51 | 50 | 13 | 5 | 0 | 2 |
| 1983-87 | 1985 | 272 | 53 | 59 | 17 | 4 | 1 | 1 |
| 1984-88 | 1986 | 331 | 64 | 56 | 17 | 3 | 3 | 0 |
| 1985-89 | 1987 | 397 | 97 | 69 | 16 | 5 | 3 | 2 |
| 1986-90 | 1988 | 480 | 126 | 93 | 19 | 4 | 4 | 2 |
| 1987-91 | 1989 | 576 | 167 | 113 | 25 | 6 | 5 | 3 |
| 1988-92 | 1990 | 622 | 189 | 122 | 25 | 6 | 4 | 4 |
| 1989-93 | 1991 | 637 | 192 | 123 | 25 | 8 | 2 | 4 |
| 1990-94 | 1992 | 611 | 176 | 115 | 24 | 6 | 3 | 2 |
| 1991-95 | 1993 | 566 | 166 | 100 | 20 | 6 | 3 | 2 |
| 1992-96 | 1994 | 528 | 144 | 85 | 15 | 4 | 2 | 1 |
| 1993-97 | 1995 | 540 | 152 | 85 | 9 | 3 | 2 | 0 |
| 1994-98 | 1996 | 585 | 170 | 98 | 7 | 1 | 3 | 0 |
| 1995-99 | 1997 | 718 | 200 | 108 | 11 | 3 | 2 | 1 |
| 1996-00 | 1998 | 880 | 226 | 120 | 15 | 5 | 2 | 1 |
| 1997-01 | 1999 | 997 | 264 | 141 | 22 | 5 | 3 | 1 |
| 1998-02 | 2000 | 1088 | 293 | 149 | 28 | 7 | 6 | 2 |
| 1999-03 | 2001 | 1157 | 307 | 149 | 34 | 9 | 8 | 3 |
| 2000-04 | 2002 | 1146 | 304 | 167 | 33 | 8 | 8 | 2 |
| 2001-05 | 2003 | 1070 | 297 | 159 | 28 | 8 | 9 | 2 |
| 2002-06 | 2004 | 1007 | 267 | 139 | 24 | 7 | 8 | 2 |
| 2003-07 | 2005 | 940 | 248 | 127 | 23 | 5 | 6 | 1 |
