# Supplementary material for: The occurrence of ‘Sleeping Beauty’ publications in medical research: Their scientific impact and technological relevance
Source: PLoS One. 2019 Oct 18;14(10):e0223373. doi: 10.1371/journal.pone.0223373 (PMC6799932; doi:10.1371/journal.pone.0223373)
Supplement: S8 Table — (DOCX) [file pone.0223373.s011.docx]

**S8 Table. Number of SBs (*s*=10) for awakening citation-intensity intervals.**

|  |  |  |  |  |  |  |
| --- | --- | --- | --- | --- | --- | --- |
|  |  | 5.0≤  ***c_a_***  ≤6.0 | 6.0<  ***c_a_***  ≤7.0 | 7.0<  ***c_a_***  ≤9.0 | 9.0<  ***c_a_***  ≤11.0 | 11.0<  ***c_a_***  ≤15.0 |
|  | s=10 |  |  |  |  |  |
| 1980-84 | 1982 | 42 | 22 | 9 | 1 | 1 |
| 1981-85 | 1983 | 56 | 31 | 12 | 0 | 1 |
| 1982-86 | 1984 | 77 | 35 | 14 | 2 | 1 |
| 1983-87 | 1985 | 88 | 37 | 15 | 2 | 2 |
| 1984-88 | 1986 | 94 | 41 | 15 | 3 | 2 |
| 1985-89 | 1987 | 86 | 38 | 11 | 5 | 3 |
| 1986-90 | 1988 | 77 | 27 | 10 | 5 | 4 |
| 1987-91 | 1989 | 63 | 25 | 7 | 3 | 4 |
| 1988-92 | 1990 | 64 | 22 | 7 | 3 | 4 |
| 1989-93 | 1991 | 58 | 17 | 7 | 2 | 3 |
| 1990-94 | 1992 | 66 | 17 | 9 | 0 | 2 |
| 1991-95 | 1993 | 73 | 21 | 11 | 1 | 1 |
| 1992-96 | 1994 | 86 | 20 | 14 | 2 | 2 |
| 1993-97 | 1995 | 100 | 25 | 15 | 3 | 1 |
| 1994-98 | 1996 | 122 | 29 | 21 | 6 | 2 |
| 1995-99 | 1997 | 136 | 31 | 26 | 6 | 3 |
| 1996-00 | 1998 | 146 | 33 | 25 | 7 | 3 |
| 1997-01 | 1999 | 145 | 33 | 22 | 8 | 4 |
| 1998-02 | 2000 | 133 | 28 | 22 | 7 | 4 |
