# Supplementary material for: The occurrence of ‘Sleeping Beauty’ publications in medical research: Their scientific impact and technological relevance
Source: PLoS One. 2019 Oct 18;14(10):e0223373. doi: 10.1371/journal.pone.0223373 (PMC6799932; doi:10.1371/journal.pone.0223373)
Supplement: Attachment — Submitted filename: MedSB-PLOS-ResponseToReviewers-20190828.docx [file pone.0223373.s012.docx]

Response to the comments and suggestions the reviewers made to our manuscript ‘PONE-D-19-15090 “*The Occurrence of ‘Sleeping Beauty’ Publications in Medical Research: Their Scientific Impact and Technological Relevance”.*

**First of all we want to thank the anonymous reviewers for their extensive comments and suggestions. Below we indicate point by point how we have dealt with the comments and suggestions.

Review Comments to the Author

Reviewer #1:

1.1 *The manuscript "The Occurrence of 'Sleeping Beauty' Publications in Medical Research: Their Scientific Impact and Technological Relevance" presents an analysis of sleeping beauties (papers with delayed recognition) in medical research fields and a subsequent analysis of citations from patents to the sleeping beauties. Herewith the authors continue their previous work on this topic. In general, the manuscript is well written and interesting. There are a few often occurring inconsistencies, e.g., awake vs. awakening, SB vs. SBs, Sleeping Beauty vs. SB, and "Fig" vs. "Fig.". Many commas after introductory phrases are missing, e.g., "In follow-up work [7, 8] we .." should read "In follow-up work [7, 8], we ..".*

**We carefully checked the manuscript to correct inconsistencies, misspellings and other errors where appropriate. In case Sleeping Beauties (SBs) are cited by a patent we call them SB-SNPRs, so the plural form comes at the end of SNPR (i.e., SB-SNPRs). PLOS requires that ‘Figure’ must be abbreviated to ‘Fig’, thus without a point.

1.2 *I see a problem with the open data policy of PLoS One and the data used in this study. I disagree with the authors' statement regarding data availability. I suggest that the authors share a small test data set (maybe from CrossRef) along with their "efficient search algorithm written in SQL" so that readers can reproduce the work in principle.*

**The algorithm depends completely on the large in-house CWTS bibliometric database (particularly specific sub-databases and calculation routines) and cannot be used without this database. Relevant data are provided in the Supporting Information file and additional data in the Open Science Platform (OSF).

1.3 *The authors should add a very recent study on a closely related subject to the introduction:*[*https://arxiv.org/abs/1906.07953*](https://arxiv.org/abs/1906.07953)*.*

**We mention this study in the text and added a reference to this article.

1.4 *The authors choose to use "a five-years awake period". As mentioned above, this probably should be "a five-years awakening period".*

**The text has been adapted.

1.5 *Furthermore, it would be good to provide a reason for this arbitrary choice.*

**The peak in the citation distribution for most medical research fields is 3-4 years after publication. So a period of 5 years covers this peak well and is a good starting point for longer sleeping periods.

1.6 *The definition of "ca" and "cs" becomes problematic with the equations, e.g., does "A.cs" mean A times cs or A times c times s? From the context, it becomes clear that the former is the case, but it would be clearer if ca and cs would have "a" and "s" as subscript.*

**In the manuscript ‘ca’ and ‘cs’ are consistently replaced by ‘c_a_’ and ‘c_s_’ (a and s in subscript)

1.7 *How were "medical research fields" defined for this study, by a list of WoS subject categories or research areas?*

**In the ‘Introduction’ section we added the text “We used the WOS categories presented in S1 Table to delineate the medical research fields …”

1.8 *The "normalization on the basis of the growth factors given in Table 2" (p. 5) should be explained in more detail. Is it simply N(s)/(growth factor)?*

**Yes, and we indicated this in the text.

1.9 *There is a strange sentence on page 7: "In this way, we are able to determine the probability that an SB occurs with specific values of the three above variables occurs." Maybe, it's fixed when the second "occurs" is removed.*

**You are right. The second “occurs” has been removed.

1.10 *Page 10: "In our 2004-study we ... ." Better provide the reference.*

**In the context of the ‘Grand Sleeping Beauty Equation’ our study in 2004 is central, and we think that mentioning it as our ‘2004-study’ this is made clear to the reader. We agree that it is better to give also the reference, and we added this reference to the publication to the text in the relevant places.

1.11 *Also page 10: "Again this has to done for each sleeping period separately ... with a different sleeping periods ... ." --> "Again this has to be done for each sleeping period separately ... with different sleeping periods ... ."*

**We have modified the text to correct this error.

1.12 *Page 14 and 20/21: "... than de sleeping time ..." --> "... than the sleeping time ..."*

**We have modified the text to correct this error

1.13 *Also page 14: "We intend to focus on these development ...". Either "We intend to focus on this development ..." or "We intend to focus on these developments ..."*

**We have modified the text to correct this error

1.14 *Page 16: "These 59 SB-SNPRs are cited by 206 patents, of which nearly half, 95, deal with inventor-author self-citation." This sounds like the 95 patents have inventor-author self-citations as a topic. Please rephrase.*

**We rephrased the text as: “These 59 SB-SNPRs are cited by 206 patents. In nearly half of these 206 patents, 95, we have inventor-author self-citation.”

1.15 *Page 18: "... ten tear ..." --> "... ten years ..."*

**We have modified the text to correct this error.

1.16 *Caption of Fig. 22: "... SN=SNPRs ..." --> "... SB-SNPRs ..."*

**We have modified the text to correct this error

1.17 *Page 19: Please define more explicitly what do mean with concepts.*

**We have now specified this in the text.

1.18 *In the case of Fig. 25 and Fig. 26, web-startable links could be provided so that the interested reader could zoom into the maps.*

**We provide in OSF the data to create the maps in the VOSviewer which enables the interested reader to zoom into the maps.

1.19 *Page 21 and also in similar occasions, it would be better to refer to "the Matthews SB-SNPR" with a formal reference or "Matthews et al.".*

**A formal reference has been added to the text.

1.20 *Unclear sentence on page 21: "It takes until 2010 before the number of citing patents and with that the technological impact of this SB-SNPR increases rapidly, in the PatStat database Spring version 2017 39 citing patents are registered, several of these patents are highly cited by other patents." Please reword, probably it is best to split the sentence.*

**We rephrased the text as: “It takes until 2010 before the number of citing patents starts to increase rapidly. In the Spring version 2016 PATSTAT database 39 citing patents are registered, several of these patents are highly cited by other patents.”

1.21 *Axis labels of all figures should be revised. Axis labels, such as "publ y", "s", "cit y", "C", and "pub y" are not helpful to readers.*

**We improved the figure labels, but also used abbreviations to avoid excessive lettering on the axis; these abbreviations are now consistent in all figures; also we mention the axis labels explicitly in the figure captions.

Reviewer #2:

2.1 *Minor issue: Fig 3 does not force the interpretation given in the first paragraph of page 6. For the case s=5 the graph would also allow for ongoing oscillations with a net increase.*

****That is possible, but we would need much more time to confirm this, or not. We changed the text by making the interpretation less certain.

2.2 *Have the authors made all data underlying the findings in their manuscript fully available? YES But with the following qualifications:The authors refer to an in-house Web of Science derived custom database, which in this form is not available to the public. But other research institutes may have comparable access to the basic data, which presuppose a subscription - and should in principle be able to reproduce the results - which are given in more numerical detail in a supplement - or even apply the methodology with another research focus.*

**In the “Materials and methods” section we documented the data sources by adding the following text: “Data sources. In this study we use the following data sources 1) the CWTS in-house enhanced Web of Science (WoS) database and 2) the Spring 2016 version of the PATSTAT database. The CWTS in-house version of the WOS is compatible as the on-line version provided by Clarivate Analytics but is limited to publications from 1980 onwards. An advantage of the in-house version of the WoS is that it is possible to cross-link information from different databases. The PATSTAT database covers information of all relevant patents worldwide and is provided by the European Patent Office especially for statistical patent analysis.”

2.3 *Minor issue: To that end it would be helpful to disclose the “fast and efficient search algorithm written in SQL”.*

****As mentioned before: the algorithm depends completely on the large in-house CWTS bibliometric database (particularly specific sub-databases and calculation routines) and cannot be used without this database. Relevant data are provided in the Supporting Information file and additional data in the Open Science Platform (OSF).

2.4 *At the end of the results section the authors zoom in on the outstanding SB in their data set and its unique properties in the set of scientific SBs cited in patents. To that end they apply analyses of co-citations and co-occurrences of keywords, using the VOSviewer.*

*Minor issue: In order to view the resp. graphs properly the authors should provide the corresponding network files.*

****We provide in OSF the data to create the maps in the VOSviewer which enables the interested reader to zoom into the maps.

2.5 *Is the manuscript presented in an intelligible fashion and written in standard English? YES, Minor issues: But it is recommended to go through the whole article and eliminate some distracting typos, grammatical and syntactical errors and incorrect references to table or figure numbers and numerical values given in figures.*

****We thoroughly checked the manuscript to correct any issues.
